# Evaluation of Corneal Deformation Analyzed with Scheimpflug Based Device in Healthy Eyes and Diseased Ones

**DOI:** 10.1155/2014/748671

**Published:** 2014-06-23

**Authors:** Michele Lanza, Michela Cennamo, Stefania Iaccarino, Carlo Irregolare, Miguel Rechichi, Mario Bifani, Ugo Antonello Gironi Carnevale

**Affiliations:** ^1^Multidisciplinary Department of Medical, Surgical and Dental Sciences, Second University of Naples, Via De Crecchio 16, 80100 Napoli, Italy; ^2^Centro Grandi Apparecchiature, Second University of Naples, Via De Crecchio 16, 80100 Napoli, Italy; ^3^Santa Lucia Eye Clinic, Via Trieste 71, 00198 Cosenza, Italy

## Abstract

This study was designed to evaluate the correlation between corneal biomechanical and morphological data in healthy eyes, eyes that underwent myopic photorefractive keratectomy (PRK), keratoconus affected eyes, and keratoconus affected eyes that underwent corneal collagen crosslinking (CCC). Complete clinical eye examination of all eyes was followed by tomographic (Pentacam, Oculus, Wetzlar, Germany) and biomechanical (Corvis ST, Oculus, Wetzlar, Germany) evaluation. Differences among Corvis ST (CST) parameters in the different groups have been performed. Linear regression between central corneal thickness (CCT), intraocular pressure (IOP), and anterior corneal curvature measured with Sim'K (KM), versus corneal deformation parameters measured with Corvis ST in the different groups, has been run using SPSS software version 18.0. We evaluated 64 healthy eyes of 64 patients with a mean refractive error of −0.65 ± 1.68 D (measured as spherical equivalent), 17 eyes of 17 patients that underwent myopic PRK for a mean refractive defect of −4.91 ± 2.05 D (measured as spherical equivalent), 16 eyes of 16 patients affected by keratconus (stage 2-3 of Amsler Classification), and 13 eyes of 13 patients affected by keratoconus that underwent CCC. Our data suggest that corneal curvature would have a greater influence on corneal deformation than CCT; in fact KM values are more strongly associated with more CST parameters both about corneal change in shape and both about the corneal ability to come back at original shape.

## 1. Introduction

**Figure 1:**
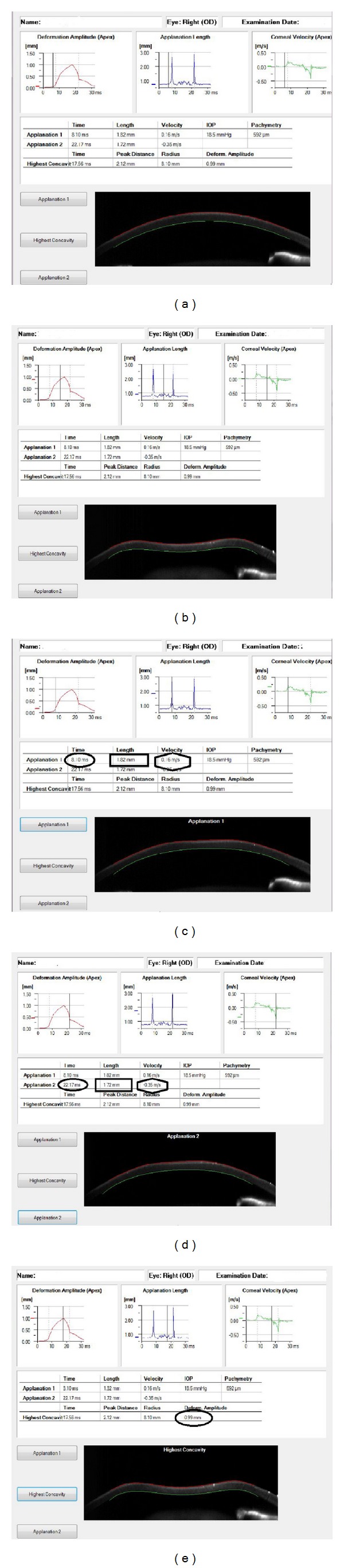
Screenshot of Corvis ST display, showing information recorded immediately upon the air impulse (a); screenshot of Corvis ST display, showing information recorded during the corneal deformation obtained by the air impulse (b); screenshot of Corvis ST display, showing Time of Applanation 1 (ellipse), Length of Applanation 1 (rectangle), Velocity of Applanation 1 (hexagon) at first applanation (c); screenshot of Corvis ST display, showing Time of Applanation 2 (ellipse), Length of Applanation 2 (rectangle), and Velocity of Applanation 2 (hexagon) at second applanation (d); screenshot of Corvis ST display, showing Deformation Amplitude at the highest concavity at corneal apex (ellipse) (e).

Until a few years ago, the corneal parameters that were traditionally studied were central corneal thickness (CCT), corneal curvature (K), and transparency, measured using different devices such as keratometers, autokeratometers, corneal topographies, corneal tomographies, slit lamps, and confocal microscopes. In 2005, Reichert introduced a new instrument, the ocular response analyzer (ORA; Reichert Ophthalmic Instrument, Depew, NY, USA), a device able to measure, in vivo, other corneal properties such as corneal hysteresis (CH) and corneal resistance factor (CRF), using a collimated air pulse to applanate the central cornea [1]. Corneal biomechanical properties measured with ORA have been widely studied in healthy subjects and in patients affected by different kinds of ocular diseases [2–16], so they have today a role in the diagnosis, follow-up, and management of many of them [7, 9, 11]. Different papers, however, showed that CH and CRF are somehow affected by corneal morphological parameters [2, 10, 13, 14, 17, 18], that is, why new kinds of technologies, like optical coherence tomography, are lately utilized in corneal biomechanical evaluation [19–21]. It would be very important to have an accurate evaluation of corneal biomechanics because it would help us in better managing alterations due to a disease (i.e., keratoconus) or to iatrogenic causes (i.e., refractive surgery); moreover, it would help in better measuring the intraocular pressure (IOP), especially in eyes affected by corneal diseases, since the current gold standard, Goldmann applanation tonometry (GAT), has been largely proven to be affected by corneal properties [6, 9, 12, 22]. The Corvis ST (Oculus, Wetzlar, Germany) (CST) is a new clinical device introduced to investigate corneal deformation properties; it uses an ultrahigh-speed Scheimpflug camera that records the deformation process in 4330 frames/sec along an 8 mm horizontal corneal coverage, while an air puff indentation causes corneal deformation (Figure 1). The ORA, instead, measures corneal shape changes with an electrooptical collimation detector system in the central 3.0 mm diameter area, throughout the 20 millisecond measurement [1].

Repeatability, reproducibility, and correlations between the parameters provided by CST have been studied by Hon and Lam [23] and by Nemeth et al. [24]; other authors evaluated CST in IOP measuring with no analysis of corneal deformation parameters [21, 25–27].

Purpose of our study is to evaluate the corneal behaviour using a Scheimpflug camera in corneas that are very different in their structure and morphology as in healthy eyes, in eyes that underwent myopic PRK, in keratoconus affected eyes, and in keratoconus affected eyes that underwent CCC. This kind of comparison has not been studied in published papers.

## 2. Materials and Methods

### 2.1. Subjects Enrolled

The study comprised 64 healthy eyes of 64 healthy subjects with a mean refractive error of −0.65 ± 1.68 D (measured as spherical equivalent), 17 eyes of 17 patients that underwent myopic PRK for a mean refractive defect of −4.91 ± 2.05 D, 16 eyes of 16 patients affected by keratoconus (KC) (stages 2 and 3 of Amsler classification) and 13 eyes of 13 patients affected by keratoconus that underwent CCC. All eyes underwent a complete ophthalmic evaluation and a corneal tomography performed using Pentacam and CST scan, and IOP evaluation with Goldmann applanation tonometry was run at last in order to not create bias in corneal evaluation. PRK and CCC patients were enrolled if they had surgery at least 1 year before. Patients with systemic and/or ocular diseases that could interfere with the corneal evaluation, such as diabetes, connective tissue disorders, dry eye, uveitis, corneal opacities, and glaucoma, were excluded from the study. Subjects wearing contact lenses were asked to stop using them at least 3 days before being evaluated. Details of different groups of patients are summarized in Table 1.

Patients that underwent PRK, enrolled in this study, did not have any complication as regression and haze and were evaluated at least 1 year after surgery, with no refractive and topographic changes from the last follow-up.

KC patients were diagnosed and staged according to Amsler classification (6 were ate stage 1, 8 were at stage 2, and 3 were at stage 3).

CCC was performed with epithelium removal and according to the Dresden Protocol [28] in patients with progressive KC (9 were at stage 2 and 7 were at stage 3 of Amsler classification); these patients were evaluated at least 1 year after treatment and were enrolled only if they did not report any complication.

We have not been able to perform a pre- and posttreatment evaluation in eyes that underwent PRK and CCC because CST has been available in our department only for 1 month.

### 2.2. Devices

The Oculus Pentacam is a corneal tomographer utilizing a rotating Scheimpflug camera, largely used by ophthalmologists, and its working principles are well known [29]. For this study the 25 images per scan option were chosen. The parameters provided by Oculus Pentacam that we evaluated in this study were CCT at pupil center and anterior corneal curvature measured with Sim'K (KM).

The Corvis ST (CST) is a noncontact tonometer that measures corneal deformation [23]; parameters included in this study were the following:

- Time of Applanation 1 (AT1): time from the start until an air puff causes the corneal flattening (first applanation) as shown in Figure 1,
- Length of Applanation 1 (AL1): length of the flattened cornea in the first applanation as shown in Figure 1,
- Velocity of Applanation 1 (AV1): velocity of corneal deformation during the first applanation as shown in Figure 1,
- Time of Applanation 2 (AT2): time from the highest concavity until cornea restores its standard curvature,
- Length of Applanation 2 (AL2): length of the flattened cornea in the second applanation as shown in Figure 1,
- Velocity of Applanation 2 (AV2): velocity of corneal deformation during the second applanation as shown Figure 1,
- Deformation Amplitude at the Highest Concavity (HCDA): maximum deformation amplitude (from the start to the highest concavity) at the corneal apex as shown in Figure 1.

Three good quality Corvis ST measurements have been taken and every scan has been performed after 5 minutes from the previous one, so as to avoid an underestimation or overestimation of the corneal biomechanical parameters. All subjects started with the Pentacam evaluation and then underwent the CST one, in order to reduce bias in morphological measurements, since the air puff could introduce errors in corneal evaluation if Scheimpflug scan is performed after it. Two different and trained physicians used the two devices (MC used Pentacam and SI used CST) and they were not aware of the results obtained by the other. Despite the fact that all patients underwent bilateral evaluation, only the right eye results were included in the statistical analysis in order to eliminate any potential intrasubject effect that may occur if both eyes of the same patient were considered.

### 2.3. Statistical Analysis

The fulfilment of the data requirements for parametric analysis (normality, homogeneity of variance) was assessed by specific tests (Kolmogorov-Smirnov, Levene). All groups were compared with one-way factorial analysis of variance (ANOVA) for each parameter, followed by post hoc test LSD for single comparison. Moreover, the correlations among KM, CCT, IOP, and corneal deformation parameters measured with CST were evaluated using parametric (Pearson) test. For all tests the level of significance was set at *P* < 0.05. All analyses were performed using SPSS software version 18.0 (IBM Corp. Armonk, New York).

The study was performed in accordance with the ethical standards stated in the 1964 Declaration of Helsinki and approved by the local clinical research ethics committee; informed consent was obtained from all subjects before examination.

## 3. Results

Age and main corneal parameters of the four groups are summarized in Table 1.

Correlation between CST and Pentacam parameters are summarized in Table 2. In particular in healthy eyes AT1 show positive correlations with pachymetry and IOP and negative ones with KM. Similar correlations with IOP and KM are also present in KC and CCC groups. Also in the PRK group correlation with IOP is positive while that with KM becomes positive. AV1 shows a positive correlation with KM and a negative one with IOP in healthy and CCC groups. AT2 shows a positive correlation with KM and a negative correlation with pachymetry and IOP in the healthy group; the positive correlation with KM and the negative one with IOP are also present in the CCC group. In the PRK and KC groups AT2 is negatively correlated with IOP values. For AL2 there is only a negative correlation with KM in the healthy group. AV2 is correlated positively with pachymetry and IOP and negatively with KM in healthy and CCC groups. Positive correlations with KM and IOP are present in the PRK group. In the KC group AV2 is negatively correlated with KM values. HCDA shows a positive correlation with KM and a negative one with pachymetry and IOP in the healthy group. Similarly positive correlations with KM and negative ones with IOP are present in KC and CCC groups while negative correlations with KM and IOP appear in the PRK group.

**Figure 2:**
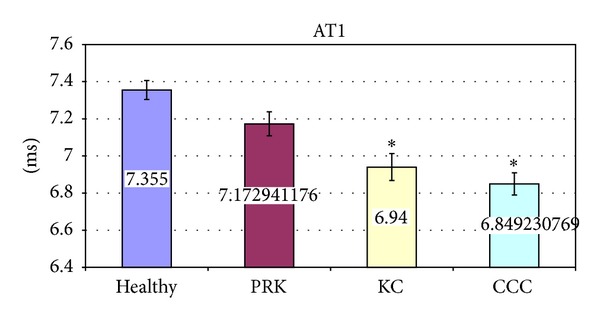
Comparison of Time of Applanation 1 (AT1) in healthy, postphotorefractive keratectomy (PRK), affected by keratoconus (KC) and postcorneal collagen cross-linking (CCC) subjects. Values are presented as mean ± standard error. Stars indicate significant differences (*P* < 0.05).

**Figure 3:**
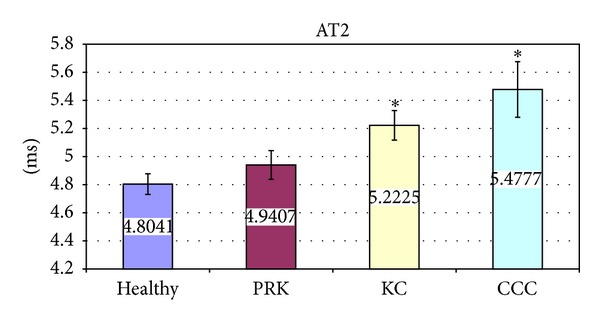
Comparison of Time of Applanation 2 (AT2) in healthy, post-PRK, KC, and post-CCC subjects. Values are presented as mean ± standard error. Stars indicate significant differences (*P* < 0.05).

**Figure 4:**
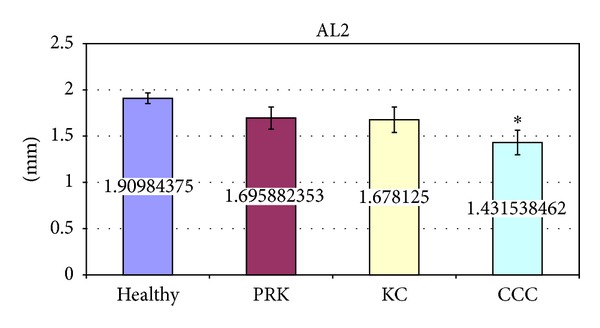
Comparison of Length of Applanation 2 (AL2) in healthy, post-PRK, KC, and post-CCC subjects. Values are presented as mean ± standard error. Stars indicate significant differences (*P* < 0.05).

**Figure 5:**
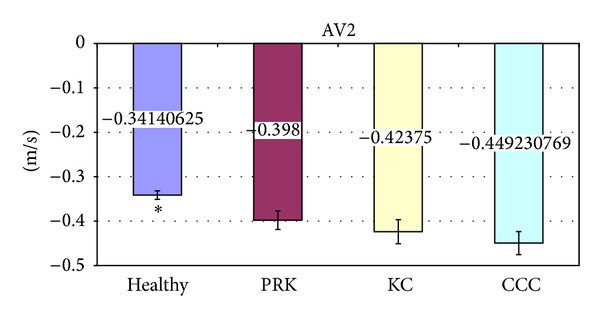
Comparison of Velocity of Applanation 2 (AV2) in healthy, post-PRK, KC, and post-CCC subjects. Values are presented as mean ± standard error. Stars indicate significant differences (*P* < 0.05).

**Figure 6:**
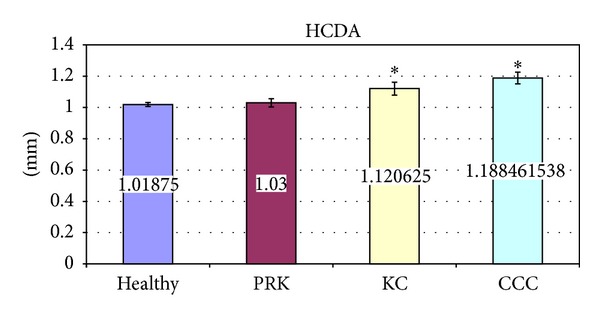
Comparison of Deformation Amplitude at the highest concavity (HCDA) in healthy, post-PRK, KC, and post-CCC subjects. Values are presented as mean ± standard error. Stars indicate significant differences (*P* < 0.05).

The significant variations of corneal deformation parameters recorded in the different groups are summarized in Table 3 and Figures 2–6. In particular IOP values in healthy and post-PRK groups were statistically higher than the ones found in KC and post-CCC groups as shown in Table 3 (one way ANOVA:* F*: 12.14,* d.f*.: 3/109, *P* < 0.000). Post hoc LSD test gave significant differences between the KC and the healthy group (−15.0%, *P* < 0.000), the CCC and the healthy group (−18.6%, *P* < 0.000), the KC and the PRK group (−9.3%, *P* < 0.047), and the CCC and the PRK group (−13.1%, *P* < 0.009). Similarly AT1 values in healthy and post-PRK groups were statistically higher than the ones found in KC and post-CCC groups as shown in Figure 2 (one way ANOVA:* F*: 11.02,* d.f*.: 3/109, *P* < 0.000). Post hoc LSD test gave significant differences between the KC and the healthy group (−5.6%, *P* < 0.000), the CCC and the healthy group (−6.9%, *P* < 0.000), and the CCC and the PRK group (−4.5%, *P* < 0.016). Conversely AT2 values in healthy and post-PRK groups were statistically lower than the ones recorded in KC and post CCC groups as shown in Figure 3 (one way ANOVA:* F*: 6.93,* d.f*.: 3/107, *P* < 0.000). Post hoc LSD test gave significant differences between the KC and the healthy group (+8.7%, *P* < 0.009), the CCC and the healthy group (+14%, *P* < 0.000), and the CCC and the PRK group (10.9%, *P* < 0.013). AL2 was significantly higher in the healthy eyes group than in the others as shown in Figure 4 (one way ANOVA:* F*: 4.28,* d.f*.: 3/109, *P* < 0.007). Post hoc LSD test gave a significant difference only between the CCC and the healthy group (−25%, *P* < 0.001). Even AV2 was significantly higher in the healthy eyes group than in others as shown in Figure 5 (one way ANOVA:* F*: 8.83,* d.f*.: 3/107, *P* < 0.000). For the healthy eyes group, post hoc LSD test gave significant differences versus PRK (−16.6%, *P* < 0.022), KC (−24.1%, *P* < 0.001), and CCC (−31.6%, *P* < 0.000). Finally HCDA in healthy and post-PRK groups was statistically lower than the one found in KC and post-CCC groups as shown in Figure 6 (one way ANOVA:* F*: 9.31,* d.f*.: 3/109, *P* < 0.000). Post hoc LSD test gave significant differences between the KC and the healthy group (+10.0%, *P* < 0.003), the CCC and the healthy group (+16.7%, *P* < 0.000), the KC and the PRK group (+8.8%, *P* < 0.031), and the CCC and the PRK group (+15.4%, *P* < 0.000).

## 4. Discussion

It is well known that the study of the biomechanical properties of the cornea is important for the diagnosis and follow-up of several ocular conditions. Many papers evaluate corneal parameters measured using ORA [1–16]. Corvis ST, the first noncontact tonometer incorporating Scheimpflug technology, has recently been introduced as a clinical device in ophthalmology to measure both IOP and corneal deformation properties [23, 24, 27].

Changes in corneal deformation are related not only to corneal structure organization and to IOP, but also to corneal biomechanics [6]; biomechanical properties measured by ORA provided information that are not always unanimous [2, 10, 13, 17, 18]. It would be very important to better understand corneal behaviour during shape modifications and corneal biomechanical properties, and this information could be used in different fields such as the following:

- having more precise values of IOP, especially in case of eyes affected by corneal disease or in ones that underwent a shape change, as happens after corneal refractive surgery,
- better understanding the evolution of corneal degenerative diseases like keratoconus, in which we observe a change both in shape and in biomechanics [4, 10],
- better screening corneas undergoing refractive surgery in order to avoid complications like ectasia.

There are limitations to this study that should be noted, first, the limited number of participants per group and we did not evaluate the corneal parameters before and after treatment as PRK and CCC but CST has been available in our department only for 1 month so we did not have time to collect these data.

AT2 values, as provided by CST, are the total of milliseconds calculated from the start of deformation until the flattened cornea rebounds from its highest concavity, reaching the second applanation.

In order to achieve a better understanding of corneal shape-changing process, we used the value obtained subtracting AT2, provided by the device, to AT1 (time from the start until an air puff causes the first corneal applanation). In this way, we obtained the time needed by the cornea to come back to a flat position after reaching the maximum deformation (HCDA) and, in our opinion, this value provides us a better idea of the time taken by the cornea to come back to its original shape after a deformation.

AT2 in fact, as you can read on CST display, is the total time from the start of the analysis, so if we had studied this parameter, our analysis about the difference between the corneal resistance to external modification and the capability of the cornea to return to its original shape after a deformation may have been biased.

According to our data, healthy and post-PRK eyes showed higher AT1 and lower AT2 compared to KC eyes and post-CCC eyes.

Corneas that are affected by KC, even if they underwent CCC, seem to be easier to applanate, compared to healthy and post PRK ones, so they show a lower resistance to deformation; moreover, it seems like they take more time to return to the applanation position and so recover the original shape. We noted with interest that corneas after PRK did not show the same values, as if the corneal thinning they underwent did not influence much their behaviour compared to healthy corneas.

It is well known that both KC and post-PRK corneas have morphological and structural differences with healthy ones; according to our data it is possible to imagine that KC induces greater changes in corneal structure that make the cornea easier to modify not only in relation to the thinning it shows; moreover, these changes prevent reaching the original shape after modifications due to external factors.

The higher deformation that KC and post CCC corneas could have is confirmed by the higher values of HCDA observed, compared with healthy and post-PRK ones.

According to our data, KM shows a significant correlation with some of the CST parameters analyzed (AT1, AT2, VA2, and HCDA) whereas CCT does not show a significant correlation in the post-PRK, KC, and post-CCC groups.

KM and CCT show a significant correlation with AT1, AT2, VA2, and HCDA in healthy corneas.

This could mean that KM influences more the deformation than corneal thickness does, in diseased corneas. This influence, however, does not seem to be the same in the four groups studied, AT1 showed values negatively correlated to KM in healthy, KC and post-CCC corneas show, indicating a higher difficulty in applanating flatter corneas whereas in eyes that underwent PRK we observed the opposite correlation.

Previous data suggest that KC and post-CCC corneas seem to be easier to modify in shape, so it is simple to imagine that the higher the corneal curvature is in healthy eyes, the less the time it takes to applanate them. In eyes after PRK, however, we observe the opposite tendency so the flatter the cornea is, the easier it is to applanate; a possible explanation is that the tissue ablation after myopic PRK makes corneas weaker to external deformations. So the greater the flattening is (meaning a higher treatment), the faster you can achieve the corneal applanation.

Interestingly, we did not observe the same correlations between CST parameters and CCT.

IOP values are directly correlated with AT1 and AV2 and inversed correlated with AV1, AT2, and HCDA in every group analyzed. Only in KC eyes AV2 is directly related but without significant value. These results mean that the resistance that IOP apply to deformation and the help that it lends in restoring the original corneal shape are effective in healthy corneas, KC ones, and ones after PRK and after CCC. The not significant value observed in AV2-IOP correlation in KC eyes could be due to two factors.

1. The small number of KC group biased the analysis.
2. IOP could not influence the corneal speed to come back at its original shape after a deformation, but this characteristic could depend from some other structural properties.

Our data support the hypothesis that corneal thinning is not the only factor that can explain the changes in corneal behavior we observe in affected corneas, and we know that biomechanic properties have an important role [1–7] but till now we could study it only with two parameters using only one device [1–7]. Values provided by CST in different groups we studied let us think that corneal deformation induced by KC (such as corneal curvature and thinning) is deeper and affects more the whole cornea making it easier to deform, compared to corneal deformation induced by PRK.

## 5. Conclusions

Although our results need to be confirmed in further studies with a larger population, they seem to be very interesting: according to our data, corneal deformation detected by CST is related much more to the corneal curvature than to the corneal thickness, especially in diseased corneas. This means that, in corneal disease screening, KM should be more important than CCT. Moreover, our study provides differences in CTS parameters in the groups analyzed and these values could be used to recognize healthy corneas, diseased ones, and borderline ones. Further studies are needed to better understand if CTS could be usefully used in clinical practice to screen eyes undergoing refractive surgery, eyes with KC at early stage, ectatic corneas, or other corneal diseases.

## Figures and Tables

**Table 1:** Mean, standard deviation (SD) and range of the parameters in the four groups evaluated in our study.

Parameters	Mean ± SD	Range
Healthy, *n* = 64		
Age (years)	35.21 ± 11.56	From 22 to 81
SE (D)	−0.65 ± 1.68	From −7.0 to 2.5
KM (D)	43.32 ± 1.26	From 40.9 to 45.9
CCT (*μ*m)	553 ± 28.51	From 498 to 631
TP (*μ*m)	551 ± 28.29	From 496 to 627
IOP (mmHg)	16.77 ± 2.31	From 13 to 24
PRK, *n* = 17		
Age (years)	32.39 ± 8.14	From 23 to 48
FU (months)	15.35 ± 1.69	From 13 to 17
KM (D)	40.26 ± 2.38	From 36.1 to 43.9
CCT pupil center (*μ*m)	448 ± 34.33	From 390 to 495
CCT thinnest (*μ*m)	447 ± 34.07	From 389 to 494
IOP (mmHg)	15.71 ± 1.72	From 13 to 19
KC, *n* = 16		
Age (years)	27.38 ± 6.09	From 19 to 37
KM (D)	47.7 ± 2.63	From 43.9 to 53.9
CCT pupil center (*μ*m)	482 ± 52.60	From 426 to 548
CCT thinnest (*μ*m)	459 ± 36.36	From 400 to 531
IOP (mmHg)	14.25 ± 1.89	From 11 to 16
CCC, *n* = 13		
Age (years)	24.44 ± 3.23	From 21 to 29
FU (months)	17.31 ± 1.11	From 15 to 18
KM (D)	47.52 ± 3.45	From 43.6 to 54.5
CCT pupil center (*μ*m)	497 ± 32.59	From 460 to 551
CCT thinnest (*μ*m)	478 ± 39.30	From 421 to 546
IOP (mmHg)	13.65 ± 1.38	From 11.5 to 16.0

SE: Spherical equivalent; FU: follow up from surgery; KM: anterior corneal curvature measured with Sim'K; CCT: central corneal thickness; TP: corneal thinnest point; intraocular pressure (IOP).

**Table 2:** Pearson's parametric correlations among CST and Pentacam parameters in the four groups evaluated in our study. In bold significant results.

		HEALTHY			PRK			KC			CCC		
		KM	CCT	IOP	KM	CCT	IOP	KM	CCT	IOP	KM	CCT	IOP
AT1	Pearson correlation	**−0.371**	**0.442**	**0.932**	**0.690**	0.354	**0.997**	**−0.609**	0.474	**0.998**	**−0.570**	0.263	**0.994**
	*P* (2-tailed)	**0.022**	**0.005**	**0.000**	**0.002**	0.163	**0.000**	**0.012**	0.064	**0.000**	**0.042**	0.386	**0.000**
	*N*	38	38	64	17	17	17	16	16	16	13	13	13

AL1	Pearson correlation	−0.089	−0.006	0.005	−0.170	0.412	−0.063	0.003	0.111	0.475	0.178	0.019	−0.458
	*P* (2-tailed)	0.597	0.970	0.969	0.515	0.101	0.810	0.991	0.682	0.063	0.561	0.951	0.116
	*N*	38	38	64	17	17	17	16	16	16	13	13	13

AV1	Pearson correlation	**0.407**	−0.151	**−0.580**	−0.184	0.356	−0.261	0.478	−0.320	0.042	**0.650**	−0.016	**−0.561**
	*P* (2-tailed)	**0.011**	0.367	**0.000**	0.479	0.160	0.311	0.061	0.227	0.878	**0.016**	0.959	**0.046**
	*N*	38	38	64	17	17	17	16	16	16	13	13	13

AT2	Pearson correlation	**0.451**	**−0.329**	**−0.580**	−0.459	−0.399	**−0.812**	0.429	−0.423	**−0.655**	**0.886**	−0.486	**−0.691**
	*P* (2-tailed)	**0.005**	**0.043**	**0.000**	0.086	0.141	**0.000**	0.097	0.103	**0.006**	**0.000**	0.092	**0.009**
	*N*	38	38	64	15	15	15	16	16	16	13	13	13

AL2	Pearson correlation	**−0.327**	0.250	−0.011	0.152	0.173	0.383	−0.351	0.262	−0.174	−0.467	0.184	0.279
	*P* (2-tailed)	**0.045**	0.130	0.930	0.561	0.507	0.129	0.182	0.327	0.518	0.108	0.547	0.355
	*N*	38	38	64	17	17	17	16	16	16	13	13	13

AV2	Pearson correlation	**−0.512**	**0.326**	**0.581**	**0.579**	−0.025	**0.728**	**−0.621**	0.374	0.258	**−0.816**	**0.584**	**0.802**
	*P* (2-tailed)	**0.001**	**0.046**	**0.000**	**0.024**	0.931	**0.002**	**0.010**	0.154	0.334	**0.001**	**0.036**	**0.001**
	*N*	38	38	64	15	15	15	16	16	16	13	13	13

HCDA	Pearson correlation	**0.541**	**−0.440**	**−0.786**	**−0.546**	−0.155	**−0.838**	**0.718**	−0.383	**−0.715**	**0.852**	−0.463	**−0.894**
	*P* (2-tailed)	**0.000**	**0.006**	**0.000**	**0.024**	0.552	**0.000**	**0.002**	0.143	**0.002**	**0.000**	0.111	**0.000**
	*N*	38	38	64	17	17	17	16	16	16	13	13	13

**Table 3:** Mean, standard deviation and range of the Corvis ST parameters in different groups evaluated in our study.

Parameters	Mean ± SD	Range
Healthy, *n* = 64		
Time of Applanation 1 (AT1) (ms)	7.36 ± 0.41	From 6.9 to 9.1
Length of Applanation 1 (AL1) (mm)	1.75 ± 0.27	From 1.3 to 2.3
Velocity of Applanation 1 (AV1) (m/s)	0.15 ± 0.04	From 0.0 to 0.2
Time of Applanation 2 (AT2) (ms)	4.80 ± 0.59	From 3.4 to 6.0
Length of Applanation 2 (AL2) (mm)	1.91 ± 0.46	From 1.0 to 2.7
Velocity of Applanation 2 (AV2) (m/s)	−0.34 ± 0.08	From −0.5 to −0.1
Deformation Amplitude at the highest concavity (HCDA) (mm)	1.02 ± 0.10	From 0.7 to 1.3
PRK, *n* = 17		
Time of Applanation 1 (AT1) (ms)	7.17 ± 0.27	From 6.9 to 7.7
Length of Applanation 1 (AL1) (mm)	1.87 ± 0.37	From 1.3 to 2.6
Velocity of Applanation 1 (AV1) (m/s)	0.17 ± 0.09	From 0.1 to 0.5
Time of Applanation 2 (AT2) (ms)	4.94 ± 0.40	From 4.2 to 5.6
Length of Applanation 2 (AL2) (mm)	1.70 ± 0.50	From 1.1 to 2.7
Velocity of Applanation 2 (AV2) (m/s)	−0.40 ± 0.08	From −0.5 to −0.3
Deformation Amplitude at the highest concavity (HCDA) (mm)	1.03 ± 0.11	From 0.8 to 1.2
KC, *n* = 16		
Time of Applanation 1 (AT1) (ms)	6.94 ± 0.29	From 6.4 to 7.3
Length of Applanation 1 (AL1) (mm)	1.66 ± 0.33	From 1.2 to 2.4
Velocity of Applanation 1 (AV1) (m/s)	0.17 ± 0.03	From 0.1 to 0.3
Time of Applanation 2 (AT2) (ms)	5.22 ± 0.42	From 4.6 to 5.9
Length of Applanation 2 (AL2) (mm)	1.68 ± 0.55	From 0.9 to 2.4
Velocity of Applanation 2 (AV2) (m/s)	−0.42 ± 0.11	From −0.6 to −0.2
Deformation Amplitude at the highest concavity (HCDA) (mm)	1.12 ± 0.16	From 0.8 to 1.5
CCC, *n* = 13		
Time of Applanation 1 (AT1) (ms)	6.85 ± 0.22	From 6.5 to 7.2
Length of Applanation 1 (AL1) (mm)	1.69 ± 0.28	From 1.3 to 2.2
Velocity of Applanation 1 (AV1) (m/s)	0.17 ± 0.04	From 0.1 to 0.2
Time of Applanation 2 (AT2) (ms)	5.48 ± 0.71	From 4.6 to 6.8
Length of Applanation 2 (AL2) (mm)	1.43 ± 0.48	From 0.9 to 2.4
Velocity of Applanation 2 (AV2) (m/s)	−0.45 ± 0.09	From −0.6 to −0.3
Deformation Amplitude at the highest concavity (HCDA) (mm)	1.19 ± 0.14	From 1.0 to 1.5
